# Non-peptidergic primary afferents are presynaptic to neurokinin-1 receptor immunoreactive lamina I projection neurons in rat spinal cord

**DOI:** 10.1186/1744-8069-8-64

**Published:** 2012-09-10

**Authors:** Abeer W Saeed, Alfredo Ribeiro-da-Silva

**Affiliations:** 1 Department of Pharmacology and Therapeutics, McGill University, Montreal, Quebec, H3G 1Y6, Canada; 2 Alan Edwards Centre for Research on Pain, McGill University, Montreal, Quebec, H3A 2B2, Canada; 3 Department of Anatomy and Cell Biology, McGill University, Montreal, Quebec, H3A 2B2, Canada; 4 Department of Pharmacology and Therapeutics, McGill University, 3655 Promenade Sir-William-Osler, Montreal, Quebec, H3G 1Y6, Canada

## Abstract

**Background:**

Pain-related (nociceptive) information is carried from the periphery to the dorsal horn of the spinal cord mostly by two populations of small diameter primary afferents, the peptidergic and the non-peptidergic. The peptidergic population expresses neuropeptides, such as substance P and calcitonin gene-related peptide, while the non-peptidergic fibers are devoid of neuropeptides, express the purinergic receptor P2X3, and bind the isolectin B4 (IB4). Although it has been known for some time that in rat the peptidergic afferents terminate mostly in lamina I and outer lamina II and non-peptidergic afferents in inner lamina II, the extent of the termination of the latter population in lamina I was never investigated as it was considered as very minor. Because our preliminary evidence suggested otherwise, we decided to re-examine the termination of non-peptidergic afferents in lamina I, in particular with regards to their innervation of projection neurons expressing substance P receptors (NK-1r). We used retrograde labeling of neurons from the parabrachial nucleus combined with lectin IB4 binding and immunocytochemistry. Samples were examined by confocal and electron microscopy.

**Results:**

By confocal microscopy, we studied the termination of non-peptidergic afferents in lamina I using IB4 binding and P2X3 immunoreactivity as markers, in relation to CGRP immunoreactivy, a marker of peptidergic afferents. The number of IB4 or P2X3-labeled fibers in lamina I was higher than previously thought, although they were less abundant than CGRP-labeled afferents. There were very few fibers double-labeled for CGRP and either P2X3 or IB4. We found a considerable number of IB4-positive fiber varicosities in close apposition to NK-1r-positive lamina I projection neurons, which were distinct from peptidergic varicosities. Furthermore, we confirmed at the ultrastructural level that there were bona fide synapses between P2X3-immunoreactive non-peptidergic boutons and neurokinin-1 receptor-positive lamina I dendrites.

**Conclusions:**

These results indicate the presence of direct innervation by non-peptidergic nociceptive afferents of lamina I projection neurons expressing NK-1r. Further investigations are needed to better understand the role of these connections in physiological conditions and chronic pain states.

## Introduction

Unmyelinated C afferents comprise approximately 70% of all primary afferents fibers [1]. The majority of such fibers transmit nociceptive information from the periphery to the spinal dorsal horn and have been divided into two main populations, the peptidergic and the non-peptidergic [2]. The peptidergic population synthesizes neuropeptides, such as substance P (SP) and calcitonin gene related peptide (CGRP), expresses the high affinity nerve growth factor receptor, trkA, and terminates mainly in lamina I and outer lamina II of the spinal dorsal horn [3-5]. The non-peptidergic unmyelinated afferents, although devoid of neuropeptides, express the purinergic P2X3 receptor, bind the plant isolectin B4 (IB4), express receptors for glial cell-derived neurotrophic factor (GDNF) and terminate mainly in inner lamina II of the spinal dorsal horn [6-8]. These two populations can also be differentiated based on the ultrastructural properties of their central terminals in the spinal dorsal horn.; The peptidergic terminate mostly as simple axo-dendritic boutons, containing several large granular vesicles (LGV), and occasionally as central terminals of type Ib glomeruli (CIb), which are rich in LGV [9,10]. In contrast, the non-peptidergic afferents terminate most often as the central bouton of type Ia glomeruli, which are poor in LGV, have a very dense matrix and are often postsynaptic to GABA-positive neurons at axo-axonic or dendroaxonic synapses [10].

Regarding peptidergic afferents, their dorsal horn termination has been investigated in particular in relation to neurons which express the SP receptor, the neurokinin-1 receptor (NK-1r) [11-13]. In particular, it has been shown at the confocal and electron microscopic levels that SP-immunoreactive (IR) terminals innervate preferentially neurons which display NK-1r immunoreactivity [11,13]. In lamina I, an abundant innervation by SP-IR primary afferents of projection neurons, which are immunoreactive for the NK-1r and are activated by noxious stimuli, has been dected [13]. Unfortunately, our knowledge concerning the central terminations of the non-peptidergic C fiber population is limited. In rat, as mentioned above, they have been shown to terminate mostly in inner lamina II as central terminals of type Ia glomeruli. However, besides the fact that they are postsynaptic to GABAergic interneurons, their synaptic connections are not well known. However, studies in rat utilizing whole-cell recordings suggest that these non-peptidergic afferents form indirect connections with lamina I projection neurons through interneurons in lamina II [14,15], while other studies, using transgenic mice, proposed that the non-peptidergic afferents synapse onto inner lamina II interneurons which in turn would connect with deep lamina V projection neurons [16]. However, it was never investigated whether lamina I projection neurons receive direct synapses from non-peptidergic afferents, likely because it was assumed that the termination of such afferents in lamina I was negligible. In this study, we examined quantitatively the innervation of NK-1r-positive lamina I projection neurons by non-peptidergic unmyelinated primary afferents, using confocal microscopy, and also provided ultrastructural evidence of direct synapses of these afferents on NK-1r-IR lamina I neurons.

## Results

### Termination of non-peptidergic afferents in lamina I

**Figure 1:**
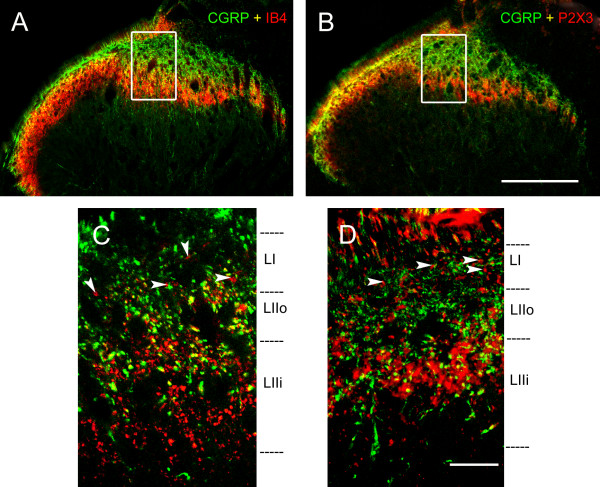
** CGRP, IB4 and P2X3 staining in transverse spinal cord sections.A** and **B** show low magnification confocal images of CGRP-IR and IB4 positive (A) or P2X3-IR (B) fibers. **C** and **D** represent high magnification confocal images from the middle third of the latero-medial extent of the superficial dorsal horn. In **C**, note that there is limited co-localization of IB4 and CGRP (in yellow). Arrowheads show axonal varicosities (boutons) from non-peptidergic fibers in lamina I, which do not co-localize CGRP immunoreactivity. The framed regions in **A** and **B** indicate the approximate regions from where **C** and **D**, respectively, were obtained (the latter originate from other sections). CGRP (in green); IB4 (in red); P2X3 (in red). Scale bar (**A**, **B**) = 200 μm; scale bar (**C**, **D**) = 20 μm.

**Figure 2:**
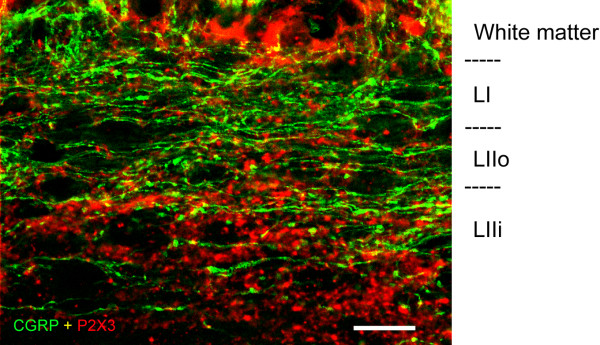
** Confocal images at high magnification obtained from parasagittal spinal cord sections showing CGRP-IR (green) and P2X3-IR (red) varicosities in the superficial dorsal horn.** P2X3-IR varicosities were present in considerable number in lamina I (LI) but were more highly concentrated in inner lamina II (LIIi). Scale bar = 20 μm.

**Figure 3:**
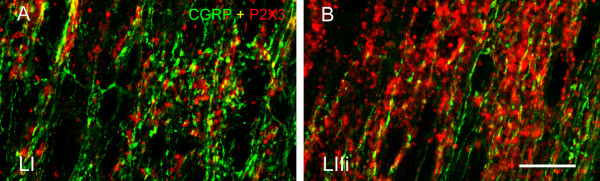
** Confocal images at high power obtained from horizontal spinal cord sections.** In a confocal optical section from lamina I adjacent to the white matter (**A**), note the relatively abundant P2X3-IR fibers with varicosities (boutons). CGRP-IR fibers and boutons were considerably more abundant in this lamina. In a confocal optical section from inner lamina II (**B**), note the very high density of P2X3-IR fibers and varicosities, higher than that of CGRP-IR fibers in lamina I. Note that most varicosities display either P2X3 or CGRP immunoreactivity, although some co-localization is observed (yellow). Scale bar (**A, B**) = 20 μm.

We examined the termination of non-peptidergic afferents using either IB4 binding or P2X3 immunoreactivity. We used sections cut in the three planes: transverse, parasagittal and horizontal (Figures 1, 2, and 3), and focused on the region equidistant from the lateral and medial limits of the dorsal horn. In transverse sections, when comparing the distribution of IB4-binding (IB4+) and P2X3-IR fibers at low magnification, it was apparent that the band of intense IB4+ labeling extended more ventrally than the P2X3 band (Figure 1). Overall, some IB4+ and P2X3-IR terminals co-localized CGRP immunoreactivity (in yellow in Figures 1 and 3), although the majority did not. A surprising observation was that the number of boutons in lamina I that were IB4+ or P2X3-IR and did not co-localize CGRP immunoreactivity was considerable, and much higher than what could be expected based on the literature. Some IB4+ and P2X3-IR boutons which did not co-localize CGRP immunoreactivity could be observed in transverse sections (Figure 1 C-D; arrowheads). However, these boutons were particularly apparent in parasagittal (Figure 2) and horizontal sections (Figure 3). In horizontal sections, we could be absolutely certain that the boutons from non-peptidergic afferents were located in lamina I since we used serial sections, and the confocal images were obtained with a very small pinhole corresponding to an optical slice of ~0.5 μm adjacent to the white matter. These sections revealed a considerable innervation of lamina I by boutons immunoreactive for P2X3, although they were clearly less abundant than the CGRP-IR (Figure 3A). The comparison with optical slices from inner lamina II allowed us to assess how much more abundant P2X3-IR boutons were in that layer (Figures 1D, 2, and 3B), confirming that inner lamina II is the main termination site for non-peptidergic afferents.

### Innervation of NK-1r-IR lamina I projection neurons by non-peptidergic afferents

**Figure 4:**
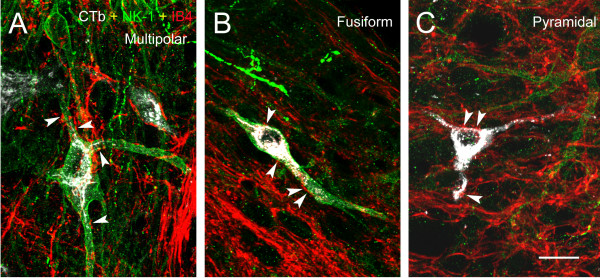
** Confocal triple-labeling image showing the innervation of lamina I spinoparabrachial (A) multipolar, (B) fusiform and (C) pyramidal neurons by IB4+ varicosities (indicated by arrowhead).** IB4 (red); NK-1r (green); spinoparabrachial neurons labeled with CTb (white). Scale bar (**A-C**) = 20 μm.

**Figure 5:**
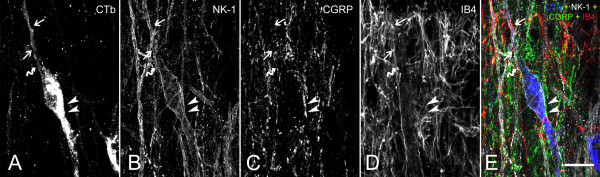
** Example of a quadruple labeling observed at the confocal level using a multi-track approach.** In this image the following signals were simultaneously detected: CGRP (green); IB4 binding (red); CTb transported retrogradely from the parabrachial nucleus (blue); NK-1r (white). A fusiform neuron, double labeled with CTb and NK-1r, is innervated by CGRP-IR boutons (arrowhead) and IB4+ (arrow) boutons, which represent distinct populations. However, a small population of varicosities co-labeled for CGRP and IB4 (curved arrow) was detected. Scale bar (**A-E**) = 20 μm.

To study the innervation of NK-1r-IR lamina I projection neurons by non-peptidergic afferents, we labeled neurons retrogradely by means of a stereotaxic cholera toxin subunit b (CTb) injection in the lateral parabrachial nucleus. The injection site covered most of the parabrachial complex, including the lateral parabrachial nucleus, an observation that is comparable to distributions previously reported by us [17,18]. The majority of the retrogradely labeled lamina I neurons were found on the contralateral side, although a few were also present on the side ipsilateral to the CTb injection. CTb labeling of spinoparabrachial lamina I neurons included the cell body and primary dendrites (Figures 4 and 5). 

We also performed a labeling of these cells with antibodies against the SP, receptor, the NK-1r. Indeed, it has been previously described [17,18] that lamina I, fusiform and multipolar spinoparabrachial neurons, identified by retrograde tracing of CTb, often express NK-1r immunoreactivity, while lamina I pyramidal neurons seldom express NK-1r immunoreactivity. These neuronal populations can be identified based on their dendritic arborization and cell body shape when viewed in the horizontal plane. Multipolar neurons have four or more dendrites arising from an irregularly-shaped soma (Figure 4A). Fusiform neurons possess two dendrites, each arising from one end of a spindle-shaped soma (Figure 4B and 5). Lastly, pyramidal neurons have a triangular soma with one primary dendrite arising from each of the three tips of the soma (Figure 4C). In this study, we observed IB4+ non-peptidergic varicosities in apparent direct apposition to all three populations of lamina I projection neurons, multipolar (Figure 4A), fusiform (Figure 4B) and pyramidal (Figure 4C). These boutons were distinct from CGRP-positive peptidergic boutons, and from boutons of the small proportion of primary afferents which are simultaneously CGRP-positive and bind IB4, as described previously [19,20] (Figure 5).

### Quantification of non-peptidergic varicosities apposed to NK-1r-positive lamina I projection neurons

**Figure 6:**
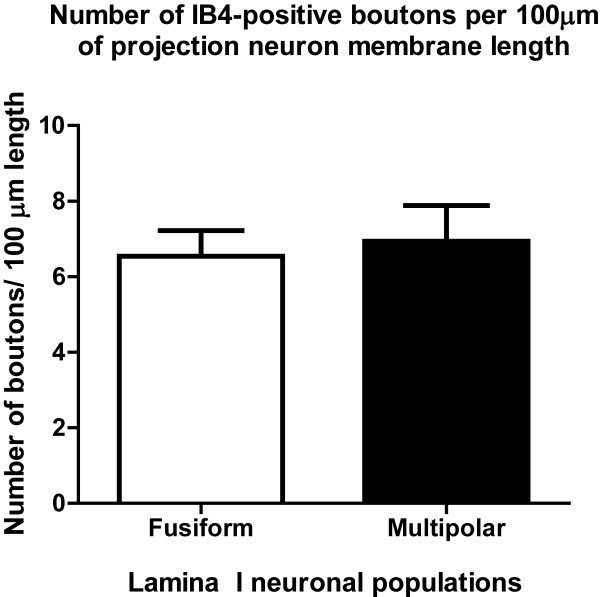
** Quantification of IB4-positive boutons in direct apposition to the cell body and primary dendrites of NK-1r-IR lamina I spinoparabrachial neurons.** Results are expressed in number of appositions from boutons per 100 μ of membrane length of NK-1r-IR cell. N = 6 animals.

As the objective of this study was to investigate the non-peptidergic innervation of NK-1r-IR projection neurons, our quantitative analysis focused on the innervation of multipolar and fusiform neurons. Indeed, pyramidal neurons are seldom immunoreactive for the substance P receptor [21]. Our quantitative analysis revealed a substantial innervation of NK-1r-IR multipolar and fusiform populations of lamina I projection neurons by non-peptidergic afferents (Figure 6). Since the main objective of this paper was to give an account of the non-peptidergic innervation, no quantification was performed for the peptidergic innervation on lamina I neurons, which has been previously investigated [11,22,23]. 

### Ultrastructural demonstration of synapses between non-peptidergic primary afferents and lamina I neurons

**Figure 7:**
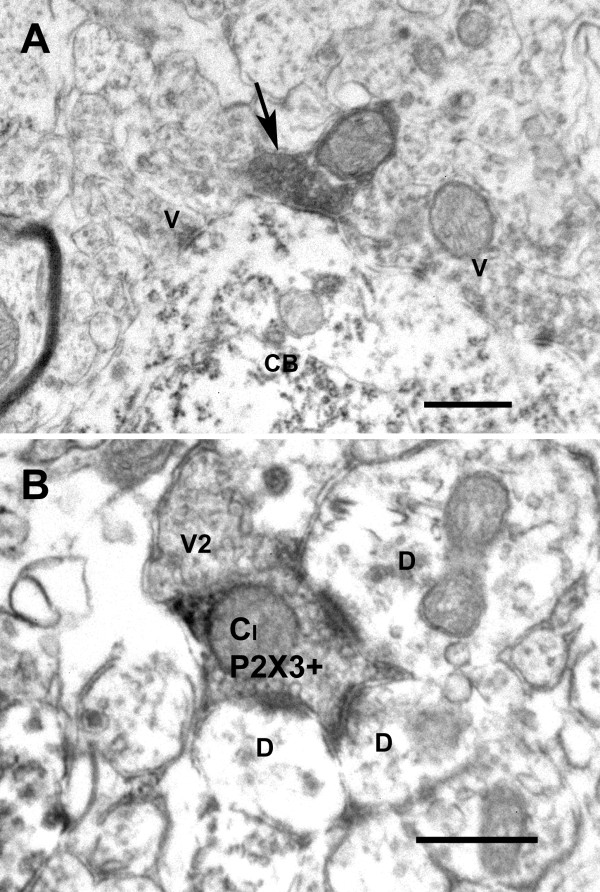
** Electron micrograph showing: in A, a P2X3-IR bouton (arrow) forming an axo-somatic contact onto a lamina I cell body (CB), which is also postsynaptic to unlabeled axonal boutons (V); in B, a P2X3-IR central bouton of a type Ia glomerulus (CI P2X3+) presynaptic to 3 lamina I dendrites (D) and apposed to an axonal bouton (V2).** Scale bar = 0.5 μm.

Because the resolution of confocal microscopy is insufficient to demonstrate the presence of bona fide appositions of two structures and synapses between them, we performed an ultrastructural study using P2X3 immunoreactivity. We had to omit glutaraldehyde from the fixative because it completely blocked the immunostaining, even in very low concentration, which affected the quality of the ultrastrucure. In lamina I, all P2X3-IR boutons established simple axo-dendritic or axo-somatic synapses (Figure 7A). In inner lamina II, most P2X3-IR boutons were involved in complex synaptic arrangements, as predicted from the literature [24], forming the central element of type Ia glomeruli (Figure 7B). 

**Figure 8:**
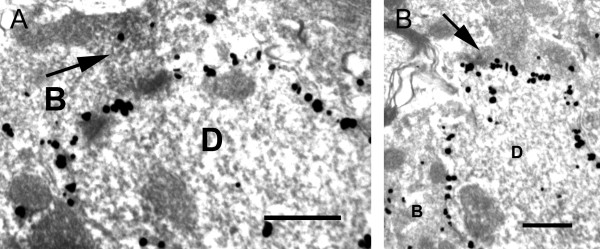
** Electron micrographs showing axo-dendritic contacts between P2X3-positive boutons (arrows) and NK-1r-positive dendrites (D) (A and B).** Note the silver-intensified gold particles along the plasma membrane of the dendrites, representing NK-1r-IR sites. **B**, unlabeled axonal bouton. Scale bar = 0.5 μm.

We used a double labeling for P2X3 and NK-1r to demonstrate direct appositions and synapses between P2X3 immunoreactive boutons and NK-1r dendritic profiles (Figure 8).

## Discussion

In this study, we demonstrate the presence in lamina I of a significant number of boutons originating from non-peptidergic afferents immunoreactive for P2X3 or binding the lectin IB4. We also provide evidence at the confocal microscopy level that IB4+ boutons are in apposition to lamina I projection neurons immunoreactive for the NK-1r. Lastly, we provide ultrastructural evidence of synapses between P2X3-IR boutons and lamina I dendritic profiles immunoreactive for the NK-1r.

### Technical considerations

Because most of this study was carried out using confocal microscopy, we could not fully ensure that boutons from non-peptidergic afferents were presynaptic to neurons in lamina I. For this reason, we carried out an ultrastructural study using antibodies against P2X3 and provided direct evidence of synapses between P2X3-IR axonal boutons and dendrites and cell bodies in lamina I. We also performed at the ultrastructural level a double labelling for P2X3 and the NK-1r that demonstrated that some of the structures innervated by these boutons expressed the NK-1r, in agreement with what was assumed from the confocal data. We could observe unequivocal evidence of synaptic contacts, in spite of the fact that we had to use a fixative without glutaraldehyde and pre-treatment of the sections with a detergent for a short period to obtain P2X3 staining at the ultrastructural level. We were unable to use IB4 binding for electron microscopy because of the bad penetration of the lectin in the tissue. Conversely, we were unable to use P2X3 immunoreactivity for the study of the innervation of the neurons at the confocal level because of antibody incompatibilities when performing the required triple labeling. It should be pointed out that Naim et al. [25] examined at the ultrastructural level ultrathin sections recut from thicker sections previously examined by confocal microscopy. Naim et al.'s study showed that immunoreactive varicosities seen in close apposition to the membrane of NK-1r-IR under the confocal microscope actually formed synapses when viewed under the electron microscope. Therefore, we are confident that the IB4+ varicosities in apposition to NK-1r-IR lamina I projection neurons should establish synapses in a high proportion of cases.

It is known that in rat there is a small proportion of peptidergic sensory fibers in lamina I and II that colocalize CGRP and somatostatin, do not respond to NGF and bind IB4 [19,20]. This obliged us to investigate the co-localization of IB4 binding and P2X3 immunoreactivity, our markers of non-peptidergic nociceptive C fibers, with CGRP immunoreactivity. Indeed, as predicted, we found a limited level of co-localization of either marker of non-peptidergic afferents with CGRP immunoreactivity. However, most varicosities that we observed in lamina I which were IB4+ or P2X3-IR did not co-localize CGRP immunoreactivity, what reassured us regarding the validity of our findings regarding the overall innervation of lamina I by non-peptidergic afferents. But it was still possible that a subpopulation of these afferents innervating NK-1r-IR lamina I projection neurons would represent exactly this minor subpopulation that is simultaneously CGRP-IR and IB4+. Therefore, we carried out a quadruple labeling in which we detected CTb (the retrograde label), NK-1r, CGRP and IB4 binding. Unfortunately, because of antibody incompatibilities, we could not perform P2X3 staining and had to use the lectin IB4 conjugated to a fluorochrome. Although our confocal microscope can identify 4 separate signals reliably using the multi-track approach, it was technically impossible to perform a quantitative analysis of the innervation of lamina I cells using more than 3 signals (IB4, CTb and NK-1r) because of photobleaching during the performace of the Z-stacks. However, we examined enough cells using the quadruple labeling to ensure that the proportion of axonal boutons colocalizing IB4 binding and CGRP immunoreactivity in apposition to lamina I neurons was low. An example of the quadruple labeling is given on Figure 4.

### Innervation of lamina I projection neurons by non-peptidergic C fibers

The main objective of this study was to investigate the issue of the innervation of NK-1r-IR lamina I projection neurons by non-peptidergic C fibers because in our knowledge a systematic study combining labeling of these sensory fibers with labeling of these lamina I neurons had never been done. This is an important issue for the reasons given below. Previous work by Lu and Perl [14], using simultaneous whole-cell recordings from pairs of neurons, provided some evidence that input from primary afferent C fibers terminating in inner lamina II may reach lamina I projection neurons via interposed interneurons. Since the great majority of C fibers innervating inner lamina II are non-peptidergic, it has been later suggested that the above pathway may be important for pain-related information conveyed by non-peptidergic C fibers to reach lamina I projection neurons which then project to supraspinal levels. If this is true, lamina I nociceptive projection neurons would receive direct input from peptidergic afferents and polysynaptic input from the non-peptidergic afferents [24].

Alternatively, it has been proposed that the non-peptidergic afferents are part of a distinct and parallel pathway from that of their peptidergic counterpart. This view obtained some support from a study by Braz et al. [16] in a transgenic mouse, which demonstrated that the termination of non-peptidergic afferents on lamina II would be on excitatory interneurons, which in turn would synapse on deep lamina V projection neurons with ascending connections to the amygdala, hypothalamus and bed nucleus of stria terminalis. As they found minimal connection with lamina I neurons expressing the NK-1r, the above group proposed the involvement of the non-peptidergic afferents in the affective/emotional component of pain. Alternatively, other studies also in the mouse, proposed that distinct subsets of primary sensory afferents selectively mediate responses to different stimulus modalities. These studies provided some evidence suggesting that, in the mouse, non-peptidergic afferents play a particular role in transmitting mechanical pain, as opposed to the peptidergic which would be involved in conveying thermal pain [26,27]. This idea of divergent pain pathways can be criticized since all the studies supporting it were performed in mice, which have been shown to demonstrate an explicit dichotomy between the C fiber populations that does not apply to rats and higher order species such as primates. In particular, the vanilloid TRPV1 receptors are localized only in the peptidergic fibers in mice [28,29] whereas they are present in both peptidergic and non-peptidergic afferents in the rat and higher species [30,31].

Our data provides evidence that non-peptidergic primary afferents establish direct connections with NK-1r-IR lamina I projections neurons, in addition to the possible indirect connections via an interposed interneuron suggested in previous studies [14]. Although the non-peptidergic primary afferent termination in lamina I may seem as a minor contribution to the lamina I synaptic circuitry when compared to their termination in inner lamina II, they may prove to have a significant role in the transmission of nociceptive signals. Indeed, the terminals of these fibers in inner lamina II represent the central bouton of type Ia glomeruli, and are involved in complex modulatory circuits involving GABAergic presynaptic inhibition [24]. A direct termination of non-peptidergic afferents on lamina I cells would bypass such modulation. Since non-peptidergic afferents have a more extensive distribution in the epidermis than peptidergic afferents [32], show larger and more sustained responses to capsaicin than peptidergic afferents [33] and signal mainly via glutamate without the presence of neuropeptide co-transmitters, the activation of NK-1r-IR lamina I projection neurons by these non-peptidergic afferents would result in a different signal transmitted than when activated by the peptidergic afferents.

Our results revealed that, on average, there were 6.7 appositions from non-peptidergic boutons per 100 μm of length of NK-1r-IR lamina I projection neuron membrane. How does this compare to the peptidergic innervation of these cells as shown in other studies? Although such comparison is not fully legitimate because of methodological differences with our study, we report it here to give the reader an idea of the order of magnitude of the two types of innervation. Indeed, Todd at al. [13] have detected a density of peptidergic innervation of 16.2 contacts/100 μm of NK-1r-IR lamina I projection neuron membrane. Therefore, the non-peptidergic innervation is of lamina I NK-1R-IR neurons is substantial, although less abundant than the peptidergic. Although the focus of this study was mainly on NK-1r-IR projection neurons in lamina I, there is also a population of NK-1r-positive neurons in deep laminae III-V, which also project supraspinally and send processes to laminae I-II [25]. These neurons have been shown to receive on average 18.8 contacts per 100 μm of dendrite length from substance P-IR boutons in these superficial layers [25]. Another study has reported that these deeper neurons receive contacts from IB4-binding non-peptidergic afferents in laminae I-II, although in much lower density (just 2 appositions per 100 μm of length of NK-1r-IR dendrite membrane) than for the peptidergic afferents [34]. These values of non-peptidergic innervation of laminae III/IV neurons are considerably lower than those we found in the current study.

## Conclusion

These results expand our previous knowledge concerning the termination in dorsal horn of non-pepdergic primary afferents by providing evidence that they terminate directly on lamina I neurons expressing the NK-1r.

## Materials & methods

The guidelines of the Canadian Council on Animal Care for the care and use of experimental animals and of the International Association for the Study of Pain were followed in all the experiments. Furthermore, all studies were previously approved by the McGill University Faculty of Medicine Animal Care Committee.

A total of 26 male Sprague Dawley rats (Charles River, Quebec, Canada) weighing 225-250 g were used for the experiments. The number of animals used and their suffering were kept to the minimum necessary for the conduction of the study. Animals were exposed to 12 hr light/dark cycle and given food and water *ad libitum*. The cages were fitted with soft bedding and a plastic tube for an enriched environment and the animals were housed in cages of four.

### Animal preparation

#### Injection of tracers

For the immunohistochemistry experiments requiring retrograde tracing, six animals were anesthetized using 5% isoflurane with oxygen and placed in a stereotaxic apparatus (David Kopf Instruments, Tujunga, CA) and stabilized with non-perforating ear bars. The coordinates for the parabrachial nucleus (rostral/caudal: -9.12; medial/lateral: -2.1; dorsal/ventral: -6.3) were calculated from the Paxinos & Watson Rat Brain Atlas [35] with Bregma as the reference point. A small hole was bored through the skull at the target point, exposing the dura mater and a glass micropipette (Wiretrol II, Drummond Scientific Company, Broomall, PA) was lowered to the stereotaxic position of the parabrachial nucleus. Two μl of 1.0% solution of CTb (List, Campbell, CA) was slowly injected into the parabrachial nucleus over a period of 20 minutes, followed by a waiting period of 10 minute before the micropipette was retracted from its position to minimize leakage of the tracer. CTb was injected seven days prior to sacrificing the animals.

### Animal perfusion

For immunohistochemistry, eighteen animals were deeply anesthetized with 0.3 ml/100 g of body weight of Equithesin (6.5 mg chloral hydrate and 3 mg sodium pentobarbital i.p.). They were then perfused through the left cardiac ventricle with perfusion buffer (for composition see [36]) for one minute, followed by 4% paraformaldehyde (PFA) in 0.1 M phosphate buffer (PB), pH 7.4, for 30 minutes. The spinal cord segments L4-L5, as well as the brains from animals injected with the retrograde tracer, were extracted and post-fixed for 1 or 2 hours, respectively, in the same fixative.

For examination under the electron microscope, eight animals were anesthetized as mentioned above, and then perfused with perfusion buffer for one minute, followed by 30 minutes perfusion with a mixture of 4% PFA and 15% picric acid in 0.1 M PB. The spinal cord segments L4-L5 were taken out and post-fixed for one hour in the same fixative.

All specimens were immersed overnight in 30% sucrose in PB at 4°C for cryoprotection.

### Tissue processing

#### Confocal microscopy

The injection site at the level of the parabrachial nucleus was examined by cutting serial, 100 μm-thick coronal sections of the relevant brain region. The dorsal aspect of the L4-L5 spinal cord segment was cut into serial, 50 μm-thick horizontal sections (n = 10), 50 μm-thick parasagittal sections (n = 4) or 50 μm-thick transverse sections (n = 4). All sections were cut using a freezing sledge microtome (Leica, Richmond Hill, Ontario) and collected as free-floating in phosphate-buffered saline (PBS) with 0.2% Triton-X 100 (PBS + T). To block unspecific staining, all spinal cord sections were incubated, for one hour, in 10% normal donkey serum (NDS) (Jackson, West Grove, PA) in PBS + T at room temperature. Subsequently, the sections were placed in primary antibodies (or conjugated lectin IB4 - see below) for 48 hours at 4°C. We used a mixture of 2 or 4 primary antibodies (each raised in a different species), or IB4, in PBS + T containing 5% NDS. Next, the sections were washed in PBS + T and then incubated in species-specific secondary antibodies that were raised in donkey and conjugated to either AlexaFluor 488, AlexaFluor 405, Rhodamine RedX or biotin. The sections were incubated in 3 different cocktails: #1) rabbit anti-CGRP at a 1:200 dilution (Sigma, St Louis, MO) and lectin IB4 conjugated to AlexaFluor 568 at a 1:200 dilution (Molecular Probes); #2) rabbit anti-CGRP and guinea pig anti-P2X3 at a 1:25,000 dilution (Neuromics, Edina, MN); #3) goat anti-CTb at a 1:5000 dilution (List Biological), rabbit anti-NK-1r at a 1:10000 dilution (Sigma, St Louis, MO), guinea pig anti-CGRP at a 1:8000 dilution (Peninsula, San Carlos, CA) and lectin IB4 conjugated to AlexaFluor 647 at a 1:200 dilution (Molecular Probes). All the sections were washed with PBS + T and then (for #1) incubated for 2 hours at room temperature with donkey anti-rabbit AlexaFluor 488; (for #2) incubated for 90 minutes in a biotin conjugated donkey anti-guinea pig IgG (Jackson Immunoresearch, West Grove, PA, 1:200). Further amplification of the P2X3 signal was achieved by treating the sections with 1 hour incubation in an avidin-biotin (A + B) complex (Vectastain Elite ABC kit, Vector Laboratories) followed by tyramide (Perkin-Elmer, Norwalk, CT, 1:75) for 7 minutes. Sections were then incubated in streptavidin conjugated to AlexaFluor 568 (Molecular Probes, Eugene, OR, 1:200) and donkey anti-rabbit AlexaFluor 488. For #3, sections wereincubated for 2 hours at room temperature with secondary antibodies: donkey anti-goat Rhodamine Red X, donkey anti-rabbit AlexaFluor 488, and donkey anti-guinea pig AlexaFluor 405. Finally, all sections were washed with PBS, mounted on gelatin-subbed slides and coverslipped with an anti-fading mounting medium (Aqua Polymount; Polysciences, Warrington, PA). Slides were stored at −4°C pending further processing.

To evaluate the injection site in the parabrachial nucleus, brainstem sections were incubated with anti-CTb antibody followed by a biotinylated donkey anti-goat IgG and streptavidin conjugated to AlexaFluor 568. They were then mounted and coverslipped as described above.

#### Electron microscopy

Spinal cord specimens from the L4-L5 region were freeze-thawed in liquid nitrogen for 30 seconds, then cut into 50 μm-thick transverse sections on a Vibratome (TPI, St. Louis, MO, USA) and collected as free-floating in PBS. To increase further antibody penetration, sections were incubated for 15 minutes in PBS + T, but all further incubations were carried out in PBS without Triton. Following a short PBS wash, the sections were incubated with 50% ethanol for 30 minutes. They were then incubated for 30 minutes in 0.5% BSA (bovine serum albumin) and subsequently incubated for one hour in 10% NDS. Sections were incubated for 48 hours, at 4°C, either with a primary antibody mixture of guinea pig anti-P2X3 at dilution 1:200,000 and rabbit anti-NK-1r at dilution 1:4000, or the guinea-pig anti-P2X3 antibody (at the same dilution) alone, in 5% NDS and 0.1% BSA.

Sections were then washed several times with PBS and then incubated for 90 minutes in a biotinylated donkey anti-guinea pig IgG antibody at dilution of 1:1000 in PBS and processed for tyramide amplification as described above. Afterwards, the sections were incubated, for one hour, in A + B enzyme complex. Labeling, for the P2X3 antibodies, was revealed using following the incubation of the section with intensified DAB (3’, 3’diaminobenzidine with 1% cobalt chloride and 1% nickel ammonium sulfate) to which 1% hydrogen peroxide was added [37]. The reaction was stopped by two washes in PBS. Sections processed for P2X3 staining were then osmicated (see below). Sections previously incubated in two primary antibodies (anti-P2X3 and anti-NK-1r) were placed for 10 minutes in washing buffer (0.8% BSA and 0.5% fish gelatin in PBS; pH 7.4) and then incubated overnight at 4°C with anti-rabbit IgG conjugated to gold particles (1.4 nm diameter, Nanoprobes, Yaphank, NY) in washing buffer, at dilution of 1:200. The following day, all sections are washed for 3 minutes in washing buffer, rinsed for 3 minutes in PBS, and then immersed for 10 minutes in 1% glutaraldehyde in PBS. The sections were washed with de-ionized water for 2 minutes and then incubated for 8 minutes with an HQ silver enhancement reagent following manufacturer's instructions (Nanoprobes, Yaphank, NY). The sections were then thoroughly rinsed with de-ionized water and then washed with 0.1 M PB.

The sections were then post-fixed in 1% osmium tetroxide in PB for 1 hour at room temperature and, subsequently, dehydrated in ascending concentrations of ethanol and propylene oxide, followed by flat-embedding the sections in Epon [37]. The Epon-embedded sections were observed at low power with a light microscope and selected areas were trimmed and re-embedded in Epon blocks. The Epon blocks were then trimmed, cut into ultrathin sections (60 nm) on a Reichert-Jung ultramicrotome (Nussloch, Germany) using a diamond knife and placed onto formvar-coated one-slot grids. Finally, sections were counterstained with uranyl acetate and lead citrate and examined using a Philips/FEI CM120 electron microscope equipped with a digital camera.

### Image acquisition and quantification

Sections were examined using a Zeiss LSM 510 confocal scanning laser microscope, equipped with Argon, HeNe1 and HeNe2 lasers, plus a Titanium-Sapphire multiphoton laser (MIRA 900 F pumped by a Verdi-V5, Coherent Canada, Mississauga, ON, Canada). We used a multi-track scanning method and appropriate filters, for the separate detections of AlexaFluor 488, streptavidin conjugated to AlexaFluor 568 or Rhodamine Red X, IB4-conjugated to AlexaFluor 647 and/or AlexaFluor 405, respectively. Images used for quantification represent serial optical sections obtained along the z-axis (z-stacks), using a 63X plan-apochromatic oil-immersion objective. Furthermore, for an unbiased representation of the images taken, all the parameters of laser power, pinhole size and image detection were kept constant for all samples. The images obtained were converted to TIFF files. Our criteria of identification and quantification of lamina I neurons have been described extensively in previous publications from our laboratory [18] (see also Results section). To calculate the density of IB4-positive boutons per unit length of neuronal membrane, the length of membrane in the soma and proximal dendrites of NK-1r-IR neurons was measured with the help of an MCID Elite Image Analysis System (Imaging Research, St.Catharines, ON, Canada) in each optical section and the number of appositions from IB4-positive boutons onto it was counted. This was done on alternate optical sections from the z-stack, to avoid counting the same boutons twice.

## Competing interests

The authors declare they have no competing interests.

## Authors’ contributions

AWS designed and performed all experimental protocols described in this manuscript as well as the writing of the initial draft of the manuscript. ARS provided supervision for data analysis, study direction, image acquisition, manuscript design and revisions. Both authors have read and approved the final draft of this manuscript.
